# Fibrinogen-to-Albumin, Platelet-to-Albumin, and Creatinine-to-Albumin Ratios in the Assessment of Preeclampsia Severity: A Retrospective Case–Control Study

**DOI:** 10.3390/metabo16100751

**Published:** 2026-10-07

**Authors:** Hüseyin Kayaalp, Şeyhmus Tunc, Ebubekir Sıddık Yılmaz, Sedat Akgöl

**Affiliations:** 1 Perinatology Unit, Department of Obstetrics and Gynecology, Ankara Bilkent City Hospital, University of Health Sciences, Ankara 06800, Turkey; drebubekiryilmaz71@gmail.com; 2 Department of Obstetrics and Gynecology, Diyarbakir Gazi Yasargil Research and Training Hospital, Diyarbakir 21070, Turkey; drseyhmustunc@gmail.com; 3 Division of Gynecologic Oncology, Department of Obstetrics and Gynecology, Diyarbakir Gazi Yasargil Research and Training Hospital, Diyarbakir 21070, Turkey; drsedatakgol@gmail.com

**Keywords:** preeclampsia, fibrinogen-to-albumin ratio, platelet-to-albumin ratio, creatinine-to-albumin ratio, disease severity

## Abstract

**Background:** Preeclampsia (PE) is a multisystem pregnancy disorder associated with endothelial dysfunction, inflammation, and coagulation abnormalities. This study evaluated the fibrinogen-to-albumin ratio (FAR), platelet-to-albumin ratio (PAR), and creatinine-to-albumin ratio (CrAR) in relation to preeclampsia severity. **Methods:** This single-center retrospective case–control study included 205 pregnant women: 104 healthy controls, 70 patients with non-severe PE, and 31 with severe PE. Clinical, obstetric, neonatal, and laboratory data were reviewed. Group comparisons, receiver operating characteristic (ROC) analysis, and logistic regression adjusted for maternal age, body mass index, and gestational age at sampling were performed. **Results:** Fibrinogen and FAR were significantly higher in severe than in non-severe PE. Fibrinogen showed an area under the curve of 0.685, with 67.7% sensitivity and 58.6% specificity at a cut-off of 5.05 g/L. FAR showed an area under the curve of 0.657, with 67.7% sensitivity and 64.3% specificity at a cut-off of 0.143. Both fibrinogen and FAR remained independently associated with severe PE after adjustment for maternal age, body mass index, and gestational age at sampling. FAR did not demonstrate superior discriminatory performance compared with fibrinogen alone. PAR did not differ significantly among groups, while CrAR was higher in preeclampsia than in controls but did not discriminate severe disease. **Conclusions:** Fibrinogen and FAR were associated with severe PE but demonstrated only modest discriminatory performance. FAR did not outperform fibrinogen alone, and its incremental value beyond fibrinogen remains uncertain. Therefore, these findings should be considered exploratory and do not support the use of FAR or fibrinogen as standalone tools for clinical screening, risk stratification, or decision-making. Further prospective, multicenter studies are needed to determine whether these markers provide clinically meaningful value within broader multimodal assessment models.

## 1. Introduction

Preeclampsia (PE) is a multifactorial and multisystem pregnancy disorder that typically develops after 20 weeks of gestation and is characterized by new-onset hypertension accompanied by maternal endothelial dysfunction, systemic inflammation, and vascular injury. Inadequate remodeling of the maternal spiral arteries, impaired placental perfusion, and subsequent placental hypoxia are considered central mechanisms in its pathogenesis [1,2]. The systemic inflammatory response associated with this process may affect coagulation, vascular permeability, and renal function, resulting in alterations in biochemical and hematological parameters such as fibrinogen, albumin, platelet count, and creatinine [3,4]. Accordingly, ratios combining inflammatory, hemostatic, and metabolic parameters have recently been proposed as potentially useful biomarkers for evaluating PE and its severity.

Fibrinogen is a positive acute-phase reactant and an essential component of the coagulation cascade, whereas albumin is a negative acute-phase protein influenced by systemic inflammation, hepatic synthesis, nutritional status, and increased vascular permeability. Therefore, the fibrinogen-to-albumin ratio (FAR) may provide an integrated measure of inflammatory- and coagulation-related alterations. Previous studies have demonstrated that elevated fibrinogen levels and decreased albumin concentrations are associated with the pathophysiological processes of PE and other pregnancy-related complications [5,6]. Furthermore, increased FAR values have been reported in pregnancies complicated by PE, threatened abortion, and preterm prelabor rupture of membranes (PPROM), suggesting that FAR may be useful in assessing disease-related inflammatory and hemostatic changes [5,6,7].

Platelets respond rapidly to endothelial injury and inflammatory stimuli and may be consumed as a result of increased coagulation activity. Alterations in platelet count and platelet indices have been reported in women with PE, particularly in those with severe disease [8]. The platelet-to-albumin ratio (PAR), which combines a hemostatic parameter with albumin as an inflammation-related marker, has been investigated as a prognostic indicator in various clinical conditions [9]. However, evidence regarding the clinical value of PAR in PE remains limited.

Creatinine is widely used as a marker of renal function, whereas albumin may reflect systemic inflammation, hepatic synthetic capacity, nutritional status, and increased vascular permeability [10,11]. Evaluating these parameters together may therefore provide a more comprehensive representation of renal and systemic alterations than either marker alone. The creatinine-to-albumin ratio (CrAR) may reflect the coexistence of impaired renal function and reduced serum albumin concentrations. Importantly, serum CrAR should not be confused with the urinary albumin-to-creatinine ratio (ACR); urinary ACR reflects urinary albumin excretion relative to urinary creatinine, whereas CrAR is calculated from serum creatinine and serum albumin concentrations. Because PE may involve both renal endothelial dysfunction and increased vascular permeability, a ratio combining serum creatinine and albumin may theoretically integrate renal impairment with systemic endothelial and inflammatory alterations. Therefore, CrAR was selected as a potentially relevant composite marker that could reflect two pathophysiological components of PE within a single index. Nevertheless, the clinical significance of serum CrAR in PE, particularly in relation to disease severity, has not been sufficiently investigated.

Considering that albumin-based ratios such as FAR, PAR, and CrAR may provide an integrated reflection of inflammatory, hemostatic, renal, and endothelial alterations, we hypothesized that these indices could be associated with PE severity. Accordingly, this study aimed to comparatively evaluate FAR, PAR, and CrAR with respect to PE severity. PAR and CrAR were assessed across healthy controls, non-severe PE, and severe PE groups, whereas FAR was evaluated specifically between the non-severe and severe PE groups because fibrinogen measurements were unavailable in healthy controls. We also evaluated the ability of these indices to discriminate severe PE and investigated their associations with disease severity using univariable and multivariable logistic regression analyses.

## 2. Materials and Methods

### 2.1. Study Population

This study was conducted in accordance with the principles of the Declaration of Helsinki. Ethical approval was obtained from the Ethics Committee of Gazi Yasargil Training and Research Hospital (Date: 25 July 2025; Decision No: 567).

Ethical approval was obtained before retrospective data extraction and analysis were initiated. This was a single-center, retrospective, case–control study conducted in the Department of Obstetrics and Gynecology of Gazi Yasargil Training and Research Hospital. Pregnant women who were examined and treated in our clinic between January 2021 and January 2023 were retrospectively reviewed. Pregnant women aged 18–45 years were included in the study. The participants were classified into three groups: the healthy control group (Group 1), the non-severe PE group (Group 2), and the severe PE group (Group 3).

In this study, the diagnosis of PE was established by reviewing the patients’ previous hospital records during treatment, in accordance with the recommendations of the American College of Obstetricians and Gynecologists (ACOG). PE was defined as new-onset hypertension (≥140/90 mmHg) developing after 20 weeks of gestation in the presence of confirmed proteinuria [12]. The diagnosis of PE with severe features was also established by reviewing the patients’ previous hospital records at the time of treatment and was defined as a systolic blood pressure of ≥160 mmHg or a diastolic blood pressure of ≥110 mmHg. In addition, regardless of blood pressure measurements, the presence of at least one of the following findings was considered sufficient for the diagnosis: platelet count <100,000/mm^3^; serum transaminase levels greater than twice the upper reference limit; persistent right upper quadrant or epigastric pain; serum creatinine level >1.1 mg/dL or an increase to twice the baseline level; pulmonary edema; new-onset headache resistant to treatment; or visual disturbances [12].

The exclusion criteria were fetal anomalies, multiple pregnancy, preexisting chronic diseases, PPROM, and a history of smoking or alcohol use. The study group consisted of women diagnosed with PE, whereas the control group consisted of low-risk pregnant women without systemic disease. The control group was selected from low-risk pregnant women evaluated at the same center during the same study period who met the same age criterion and relevant exclusion criteria. No formal individual matching procedure was performed. Nevertheless, the groups were comparable with respect to maternal age, parity, and body mass index (BMI). Systemic diseases included neurological, cardiac, thoracic, gastrointestinal, genitourinary, and endocrine disorders, including diabetes mellitus (DM).

### 2.2. Data Collection

The patients’ clinical and demographic data were retrospectively reviewed. Maternal age, gravidity, parity, BMI, systolic and diastolic blood pressure, spot urine protein level, 24-h urinary protein level, mode of delivery, and gestational age at delivery were recorded. Birth weight, umbilical cord blood pH, neonatal 1- and 5-min Apgar scores, and admission to the neonatal intensive care unit (NICU) were also recorded. The patients’ laboratory test results were retrospectively obtained from previous hospital records.

For patients with PE, only laboratory results from blood samples obtained during the hospital admission at which the diagnosis of PE was established, after diagnosis and before the initiation of treatment, were included in the analysis. After diagnosis, patients with preeclampsia were managed according to disease severity and maternal and fetal clinical findings, including antihypertensive therapy, magnesium sulfate prophylaxis when indicated, antenatal corticosteroid administration when appropriate, and timing of delivery based on maternal and fetal status. Laboratory measurements obtained at other time points during routine antenatal follow-up were not used. Platelet count, creatinine, albumin, aspartate aminotransferase (AST), alanine aminotransferase (ALT), and fibrinogen levels were recorded. Because fibrinogen was not routinely measured during antenatal evaluations in all patients, fibrinogen data were unavailable for the control group. All biochemical and hematological analyses were performed in the clinical laboratory of our hospital using methods compliant with ISO 15189 quality standards [13].

The fibrinogen-to-albumin ratio (FAR), platelet-to-albumin ratio (PAR), and creatinine-to-albumin ratio (CrAR) were calculated using laboratory results obtained during the same period. FAR was calculated by dividing the fibrinogen level by the albumin level, PAR by dividing the platelet count by the albumin level, and CrAR by dividing the creatinine level by the albumin level. The following formulas were used:

FAR = fibrinogen (g/L)/albumin (g/L)

PAR = platelet count (10^9^/L)/albumin (g/L)

CrAR = creatinine (mg/dL)/albumin (g/L)

All calculations were performed using IBM SPSS Statistics version 25.0 (IBM Corp., Armonk, NY, USA).

### 2.3. Statistical Analysis

All statistical analyses were performed using IBM SPSS Statistics version 25.0 (IBM Corp., Armonk, NY, USA). The normality of continuous variables was assessed using the Kolmogorov–Smirnov and Shapiro–Wilk tests, together with visual inspection of histograms. As the continuous variables were not normally distributed, they were presented as median and interquartile range (IQR). Categorical variables were expressed as numbers and percentages.

Overall comparisons of continuous variables among the control, non-severe PE, and severe PE groups were performed using the Kruskal–Wallis test. Pairwise comparisons between the groups were conducted using the Mann–Whitney U test. For variables with three pairwise comparisons, a Bonferroni-adjusted significance threshold of *p* < 0.0167 (0.05/3) was applied to control for multiple comparisons. Categorical variables were compared using the Pearson chi-square test or Fisher’s exact test, as appropriate.

Receiver operating characteristic (ROC) curve analysis was performed among patients with preeclampsia to evaluate the ability of laboratory parameters and derived indices to discriminate severe PE from non-severe PE. The area under the curve (AUC), 95% confidence interval (CI), sensitivity, specificity, positive predictive value (PPV), negative predictive value (NPV), positive likelihood ratio (LR+), negative likelihood ratio (LR−), and optimal cut-off values were calculated. Cut-off values were reported for parameters showing statistically significant ROC performance.

Univariable logistic regression analysis was performed to examine the associations of fibrinogen, FAR, and CrAR with severe PE. FAR and CrAR were rescaled to facilitate interpretation of the regression estimates; FAR × 100 corresponds to a 0.01-unit increase in the original FAR value, whereas CrAR × 1000 corresponds to a 0.001-unit increase in the original CrAR value. Separate multivariable logistic regression models were subsequently constructed for fibrinogen, FAR, and CrAR, with adjustment for maternal age, body mass index, and gestational age at sampling. Maternal age, BMI, and gestational age at sampling were included as clinically relevant covariates with potential confounding effects on the associations between the investigated biomarkers and severe PE. Given the limited number of severe PE cases, the number of covariates included in the multivariable models was intentionally restricted to reduce the risk of overfitting. The results were reported as odds ratios (ORs) or adjusted odds ratios (aORs) with 95% CIs. A two-sided *p* value < 0.05 was considered statistically significant for overall comparisons and other analyses unless otherwise specified.

## 3. Results

A total of 205 pregnant women were included in the study. Of these, 104 constituted the healthy control group and 101 had PE. The PE group was further divided into 70 patients with non-severe PE and 31 patients with severe PE.

No statistically significant differences were observed among the control, non-severe PE, and severe PE groups with respect to maternal age, parity, or BMI (all *p* > 0.05). In contrast, gestational age at sampling differed significantly among the groups (*p* = 0.004), with lower values observed in the severe PE group. Systolic and diastolic blood pressure and spot urine protein levels also differed significantly among the three groups (all *p* < 0.001). Among patients with PE, 24-h urinary protein levels were significantly higher in the severe PE group than in the non-severe PE group (*p* = 0.008).

Gestational age at delivery, birth weight, and 1- and 5-min Apgar scores differed significantly among the groups (all *p* < 0.001). Umbilical cord pH also showed an overall difference among the three groups (*p* = 0.013), although the pairwise comparisons were not statistically significant. Neonatal intensive care unit admission rates differed significantly among the groups (*p* < 0.001), whereas the mode of delivery did not differ significantly (*p* = 0.398) (Table 1).

Overall three-group comparisons showed statistically significant differences in ALT (*p* = 0.001), AST (*p* = 0.007), platelet count (*p* = 0.023), creatinine (*p* = 0.002), and albumin (*p* < 0.001). ALT and AST levels were significantly higher in both PE groups than in the control group, whereas no significant difference was found between the non-severe and severe PE groups. Platelet count and creatinine levels differed significantly between the severe PE and control groups but did not differ significantly between the two PE subgroups. Albumin levels were significantly lower in both PE groups than in the control group. Fibrinogen levels were significantly higher in the severe PE group than in the non-severe PE group (*p* = 0.003) (Table 2).

FAR values differed significantly between the non-severe and severe PE groups (*p* = 0.012). CrAR values showed a significant overall difference among the three groups (*p* < 0.001) and were significantly different in both preeclampsia subgroups compared with the control group (*p* < 0.05); however, no significant difference was observed between the non-severe and severe PE groups (*p* = 0.680). PAR values were similar both across the three groups and in the pairwise group comparisons (*p* > 0.05) (Table 2).

**Figure 1:**
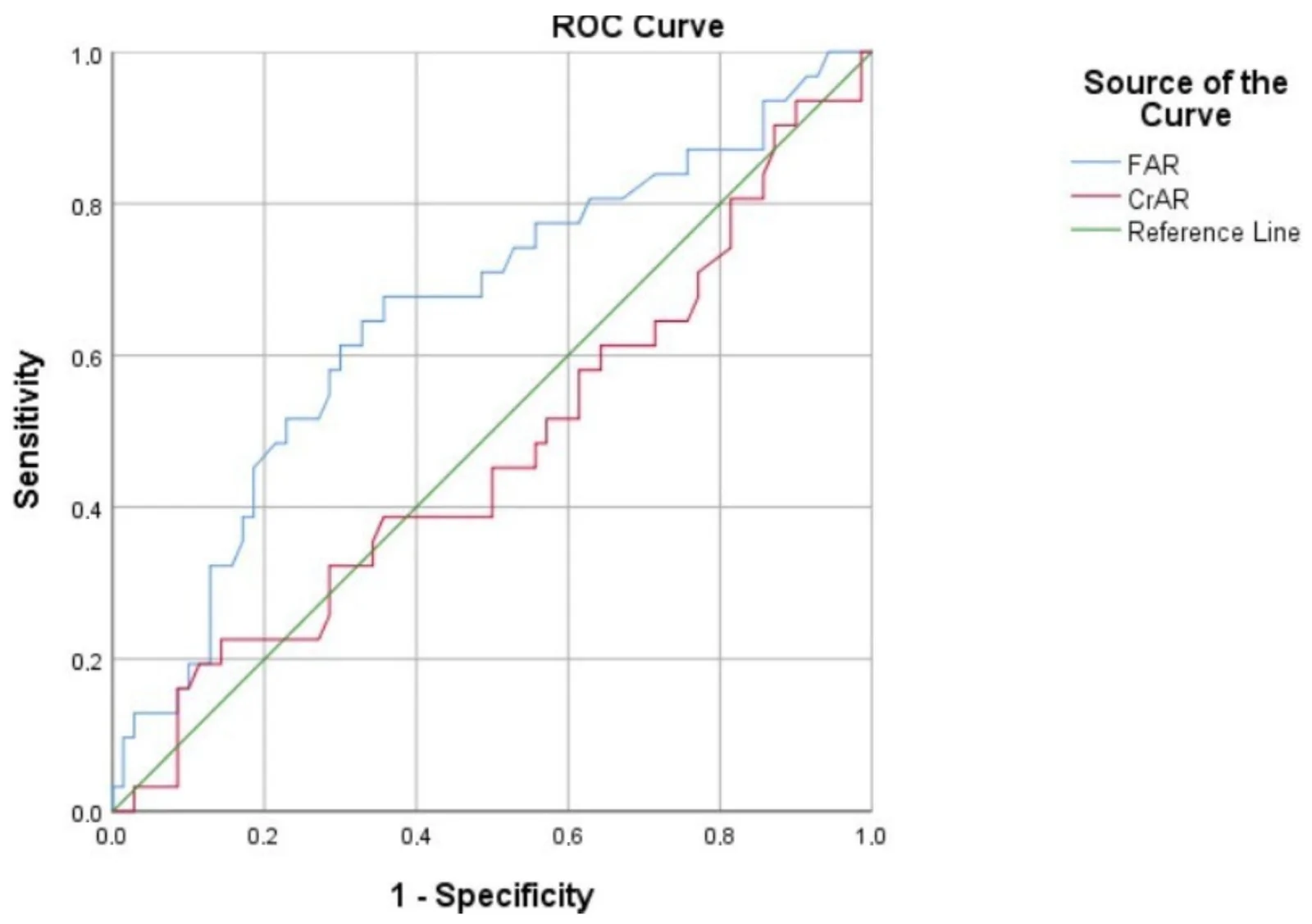
Receiver operating characteristic curves of FAR and CrAR for discriminating severe PE.

**Figure 2:**
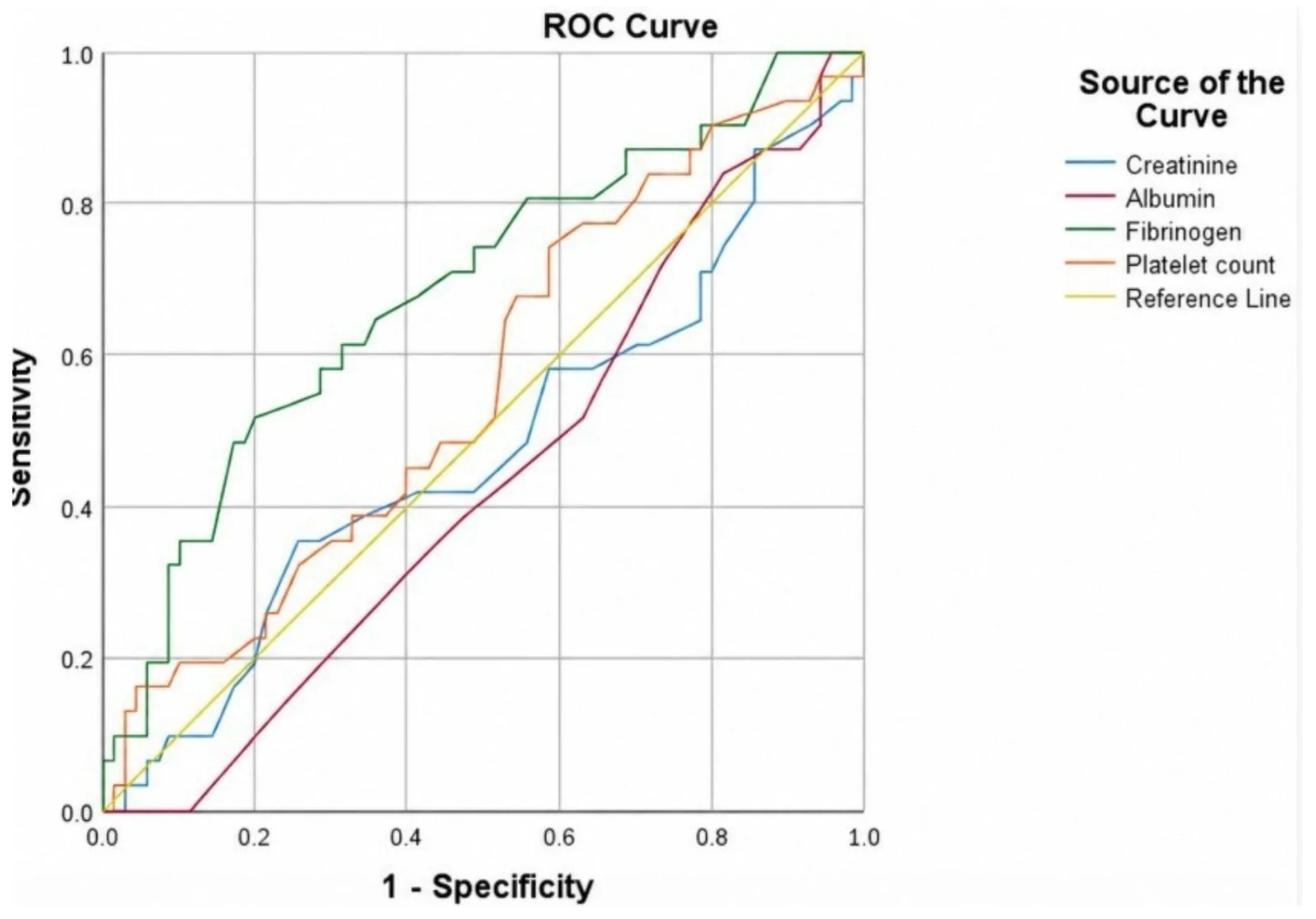
Receiver operating characteristic curves of creatinine, albumin, fibrinogen, and platelet count for discriminating severe PE.

ROC curve analysis comparing the severe PE and non-severe PE groups was performed to assess the discriminatory performance of the laboratory parameters and derived indices for severe PE. Fibrinogen showed statistically significant discriminatory performance, with an AUC of 0.685 (95% CI: 0.571–0.800; *p* = 0.003). At a cut-off value of 5.05 g/L, fibrinogen had a sensitivity of 67.7% and a specificity of 58.6%. FAR also showed statistically significant, although limited, discriminatory performance, with an AUC of 0.657 (95% CI: 0.539–0.776; *p* = 0.012). At a cut-off value of 0.143, FAR had a sensitivity of 67.7% and a specificity of 64.3%. Platelet count, albumin, creatinine, and CrAR did not show statistically significant discriminatory performance for severe PE (Table 3 and Figure 1 and Figure 2).

In the univariable logistic regression analysis, fibrinogen was significantly associated with severe PE (OR = 2.220; 95% CI: 1.301–3.787; *p* = 0.003). FAR was also significantly associated with severe PE; each 0.01-unit increase in the original FAR value was associated with a 20.3% increase in the odds of severe PE (OR = 1.203; 95% CI: 1.044–1.386; *p* = 0.011). CrAR was not significantly associated with severe PE (OR = 0.976; 95% CI: 0.878–1.085; *p* = 0.654) (Table 4).

In separate multivariable logistic regression models adjusted for maternal age, BMI, and gestational age at sampling, fibrinogen remained independently associated with severe PE (aOR = 2.242; 95% CI: 1.259–3.992; *p* = 0.006). FAR also remained independently associated with severe PE, with each 0.01-unit increase corresponding to an aOR of 1.193 (95% CI: 1.025–1.389; *p* = 0.023). CrAR was not independently associated with severe PE after adjustment for maternal age, BMI, and gestational age at sampling (aOR = 0.930; 95% CI: 0.831–1.041; *p* = 0.208). Gestational age at sampling was significantly associated with severe PE in all three multivariable models, whereas maternal age and BMI were not statistically significant (Table 5).

## 4. Discussion

This study aimed to examine differences in FAR, PAR, and CrAR values among healthy controls and patients with non-severe and severe PE, and to evaluate the discriminatory performance of these indices for severe PE.

In our study, gestational age at delivery, birth weight, and Apgar scores were lower in the PE groups, whereas NICU admission was more frequent. Fibrinogen and FAR levels were significantly higher in the severe PE group. In ROC analysis, fibrinogen and FAR showed significant discriminatory performance for severe PE; both were significantly associated with severe PE in univariable analyses and remained independently associated with severe PE in multivariable analyses. CrAR and PAR did not show significant discriminatory performance for severe PE.

The biological rationale for these composite indices may be understood within the pathophysiological framework of PE. Placental hypoxia and ischemia contribute to systemic endothelial dysfunction and a proinflammatory state, which may activate hepatic acute-phase responses and increase fibrinogen synthesis. At the same time, enhanced coagulation activation and platelet consumption may alter circulating platelet levels, while increased vascular permeability and endothelial injury may contribute to lower serum albumin concentrations. Renal endothelial involvement and changes in glomerular filtration may also influence serum creatinine levels. Therefore, FAR, PAR, and CrAR may reflect different but interconnected components of the systemic metabolic, inflammatory, coagulative, and renal alterations associated with PE [1,2,3,4,14,15,16,17].

Fibrinogen is a positive acute-phase reactant that plays a central role in the coagulation cascade. Albumin is a negative acute-phase protein influenced by inflammatory status, nutritional condition, and overall systemic health. Activation of the coagulation system by inflammatory cytokines may increase fibrinogen levels, whereas oxidative stress and endothelial injury may reduce serum albumin concentrations by increasing vascular permeability [3,14,15,16,17]. By combining these two parameters, the fibrinogen-to-albumin ratio may reflect the balance between inflammation and coagulation [7].

Previous obstetric studies have reported that FAR is associated with adverse pregnancy outcomes and preeclampsia. In a study of PPROM, FAR showed strong discriminatory performance for miscarriage (AUC = 0.840, sensitivity 76.2%, specificity 85.7%) [7]. Elevated FAR levels have also been associated with adverse outcomes in pregnancies complicated by threatened abortion [5]. In PE, previous studies have reported higher FAR values compared with healthy controls and suggested an association with disease severity [6,18]. Beyond pregnancy-related disorders, elevated FAR has also been associated with abnormal blood pressure patterns in hypertensive patients, supporting its potential role as an integrated marker of inflammatory and hemostatic burden [19].

In our study, fibrinogen and FAR levels were significantly higher in the severe preeclampsia group than in the non-severe PE group. In ROC analysis, both fibrinogen and FAR showed statistically significant but limited discriminatory performance for severe PE. Among the three investigated composite indices, FAR was the only index that differed significantly between the non-severe and severe PE groups and demonstrated statistically significant discriminatory performance for severe PE. In contrast, PAR and CrAR did not show significant discriminatory performance for severe disease. Although FAR combines fibrinogen and albumin and may reflect both hemostatic and inflammatory alterations, fibrinogen alone showed a numerically higher AUC than FAR in our study. Therefore, our findings do not support the superiority of FAR over fibrinogen, and its incremental value beyond fibrinogen was not established in the present study. Whether FAR provides any incremental clinical value beyond fibrinogen alone remains uncertain and requires direct evaluation in larger prospective studies. The novelty of the present study therefore lies primarily in the head-to-head evaluation of FAR, PAR, and CrAR within the same PE cohort, which allowed direct comparison of their relative discriminatory performance and demonstrated that only FAR showed a significant association with severe disease, while also confirming that FAR did not outperform fibrinogen alone. The relatively low AUC values further indicate that the clinical utility of these markers is limited. Given the modest AUC, sensitivity, and specificity observed for FAR, the present findings do not support its use as a standalone tool for clinical screening, risk stratification, or decision-making. Rather, any potential role of FAR should be considered exploratory and would require evaluation within broader clinical and laboratory assessment models. Furthermore, both parameters were significantly associated with severe PE in univariable analyses and remained independently associated with severe PE in multivariable logistic regression analyses adjusted for maternal age, BMI, and gestational age at sampling. These findings indicate that fibrinogen and FAR are associated with PE severity; however, their clinical utility remains uncertain. However, the absence of fibrinogen measurements in the control group and the limited discriminatory performance should be considered when interpreting these findings.

Although the exact etiology of PE remains unclear, maternal vascular and endothelial injury is known to play an important role in the development of placental insufficiency [1,2]. Endothelial injury may increase platelet consumption by promoting contact between circulating platelets and the damaged vascular surface and activating the coagulation system [4]. Previous studies have reported lower platelet counts in women with PE than in normotensive pregnant women [8,20]. In our study, platelet counts differed significantly among the three groups and were significantly lower in the severe PE group than in the control group. However, no significant difference was observed between the non-severe and severe PE groups. This finding suggests that platelet consumption may become more evident in more severe forms of the disease.

Increased vascular permeability secondary to endothelial injury may contribute to reduced serum albumin levels [6,18]. The platelet-to-albumin ratio is a composite hematological index that combines platelet count and albumin concentration. Previous studies have associated elevated PAR values with diabetic nephropathy in patients with type 2 diabetes mellitus and with prognosis in patients with malignancy [9,21]. In our study, although albumin levels were significantly lower in both PE groups than in the control group, PAR values were similar among the three groups and in all pairwise comparisons. This may be explained by simultaneous changes in platelet and albumin levels, which could reduce the discriminatory ability of the ratio. In addition, platelet count and albumin may be influenced by different and sometimes opposing physiological and disease-related processes, which may attenuate changes in their ratio. This may partly explain why PAR did not differ significantly among the groups and showed limited value for discriminating severe PE in our cohort. Our findings suggest that PAR is not a useful marker for discriminating severe PE. To our knowledge, data regarding the clinical significance of PAR in pregnancy-related conditions remain limited.

Creatinine is a metabolite that is continuously produced in the body and primarily excreted by the kidneys. Impaired renal vascular integrity and renal dysfunction may reduce creatinine excretion and consequently increase serum creatinine levels [10,11]. In the PE literature, the urinary albumin-to-creatinine ratio (ACR) has been evaluated particularly frequently. Studies conducted in Australia and India reported significantly higher ACR values in women with PE than in controls and demonstrated significant discriminatory performance for PE [22,23].

In our study, the serum CrAR was evaluated instead of urinary ACR. CrAR values differed significantly among the three groups and were significantly higher in both the non-severe and severe PE groups than in the control group. However, no significant difference was observed between the non-severe and severe PE groups. In ROC analysis, CrAR did not show significant discriminatory performance for severe PE (AUC = 0.474; *p* = 0.680). Similarly, CrAR was not significantly associated with severe PE in either univariable or multivariable logistic regression analyses.

These findings suggest that CrAR may reflect biochemical changes associated with the presence of PE but may not be sufficient for distinguishing disease severity. The clinical significance of CrAR should be further evaluated in larger, multicenter studies.

Because platelet count and serum creatinine are also components of the diagnostic criteria for PE with severe features, PAR and CrAR are potentially subject to criterion contamination. Our intention in evaluating these ratios was not to propose them as independent prognostic tools, but to explore whether their combination with albumin could provide additional information regarding the broader biochemical burden associated with severe PE. Notably, none of the patients in the severe PE group had a serum creatinine level >1.1 mg/dL or a platelet count <100,000/mm^3^. Thus, severe PE in our cohort was not characterized by renal dysfunction or thrombocytopenia according to these laboratory thresholds. Consistent with this, serum creatinine and platelet count did not differ significantly between the non-severe and severe PE groups. This clinical profile may partly explain why neither PAR nor CrAR demonstrated significant discriminatory performance for severe PE, suggesting limited added value for severity assessment beyond the underlying laboratory parameters and established clinical criteria.

The main strengths of this study were the evaluation of healthy controls, patients with non-severe PE, and patients with severe PE at the same center; the simultaneous assessment of FAR, PAR, and CrAR within the same study population; and the comprehensive investigation of their associations with severe PE using group comparisons, ROC curve analysis, and univariable and multivariable logistic regression models. In addition, restricting the analyses to blood samples obtained during the admission at which PE was diagnosed and before the initiation of treatment minimized potential variability related to treatment exposure and differences in the timing of laboratory assessment.

The main limitations of this study were its single-center, retrospective design, the limited overall sample size, and particularly the relatively small number of patients with severe PE. The relatively small number of patients with severe PE may have limited the stability and precision of the regression estimates. In addition, the relatively wide 95% confidence intervals observed in some models raise the possibility of overfitting, particularly given the limited number of severe PE cases. Therefore, the multivariable regression findings should be interpreted cautiously and require confirmation in larger cohorts. In addition, because platelet count and serum creatinine are included among the diagnostic criteria for PE with severe features, the evaluation of PAR and CrAR may be affected by criterion contamination, limiting conclusions regarding their independent value for severity assessment. Early-onset and late-onset PE may differ in their pathophysiological mechanisms and clinical characteristics, and these differences may influence biomarker profiles. Because of the retrospective design and limited sample size, particularly in the severe PE group, subgroup analyses according to the timing of disease onset could not be performed. This should be considered a limitation, and future studies should evaluate these biomarkers separately in early-onset and late-onset preeclampsia. Moreover, the absence of fibrinogen measurements in the control group prevented comparisons of fibrinogen and FAR across all three groups. Because fibrinogen was not available in the control group, the present study cannot determine whether elevated fibrinogen and FAR are associated with the presence of PE itself or only with disease severity among affected patients. This limitation applies specifically to fibrinogen and FAR; PAR and CrAR were available across all three study groups. This represents an important limitation of the study and should be considered when interpreting these findings. The retrospective collection of data may also have resulted in missing information for some obstetric and clinical variables, and the possibility of residual confounding by unmeasured factors could not be completely excluded. Finally, the single-center setting may limit the generalizability of the findings to other populations.

## 5. Conclusions

In our study, fibrinogen and FAR showed statistically significant but limited discriminatory performance for severe PE and remained associated with severe PE after adjustment for age, BMI, and gestational age at sampling. In contrast, PAR and CrAR did not demonstrate significant discriminatory performance for severe PE. FAR did not outperform fibrinogen alone, and its incremental value beyond fibrinogen was not established. Accordingly, these findings should be considered exploratory and do not establish clinical utility for any of the investigated ratios. Larger prospective, multicenter studies with direct comparative analyses are required to determine whether these indices provide additional value beyond established clinical and laboratory assessment.

## Figures and Tables

**Table 1:** Comparison of clinical and demographic characteristics among the control, non-severe PE, and severe PE groups.

	Group 1 (Control) (n = 104) Median (IQR)	Group 2 (Non-Severe PE) (n = 70) Median (IQR)	Group 3 (Severe PE) (n = 31) Median (IQR)	*p*-Value ^a^	*p*-Value ^b^	*p*-Value ^c^	*p*-Value ^d^
Age (years), Median (IQR)	27.0 (6.0)	27.9 (8.0)	27.0 (6.0)	0.634	0.366	0.576	0.882
Parity, Median (IQR)	1.0 (2.0)	1.0 (2.0)	1.0 (3.0)	0.380	0.208	0.343	0.845
BMI (kg/m^2^), Median (IQR)	28.0 (5.3)	27.0 (5.1)	26.8 (3.4)	0.392	0.198	0.415	0.799
Systolic blood pressure (mmHg), Median (IQR)	115.0 (9.0)	141.0 (8.0)	148.0 (14.0)	<0.001	<0.001	<0.001	0.001
Diastolic blood pressure (mmHg), Median (IQR)	68.0 (8.0)	90.0 (5.0)	95.0 (9.0)	<0.001	<0.001	<0.001	<0.001
Gestational age at sampling (weeks), Median (IQR)	32.5 (3.7)	32.0 (3.8)	31.0 (5.0)	0.004	0.954	<0.001	0.009
Spot urine protein (mg/dL), Median (IQR)	0.0 (0.0)	2.0 (1.2)	3.0 (1.0)	<0.001	<0.001	<0.001	<0.001
24-h urinary protein, Median (IQR)	—	930.0 (465.0)	1465.0 (820.0)	—	—	—	0.008
Gestational week at delivery, Median (IQR)	38.2 (1.0)	37.0 (3.0)	37.1 (1.0)	<0.001	<0.001	<0.001	0.157
Birth weight (g), Median (IQR)	3300.0 (512.0)	2770.0 (735.0)	2590.0 (580.0)	<0.001	<0.001	<0.001	0.947
1-min Apgar, Median (IQR)	8.0 (0.0)	7.0 (1.0)	7.0 (1.0)	<0.001	<0.001	<0.001	0.698
5-min Apgar, Median (IQR)	9.0 (0.0)	9.0 (1.0)	8.0 (1.0)	<0.001	<0.001	<0.001	0.778
Cord pH, Median (IQR)	7.34 (0.1)	7.31 (0.1)	7.33 (0.1)	0.013	0.489	0.066	0.384
NICU (n/%)	10 (9.6)	22 (31.4)	12 (38.7)	<0.001	—	—	0.475
C/S (n/%)	51 (49.0)	33 (47.1)	19 (61.3)	0.398	—	—	0.189

NICU: Neonatal intensive care unit, IQR: Interquartile Range, PE: preeclampsia, C/S: Cesarean Section, BMI: Body mass index. Statistical analysis: Overall comparisons among the three groups were performed using the Kruskal–Wallis test, and pairwise comparisons were performed using the Mann–Whitney U test. Categorical variables were compared using the Pearson chi-square test or Fisher’s exact test, as appropriate. A *p*-value < 0.05 was considered statistically significant for overall comparisons. For the three pairwise comparisons of continuous variables, statistical significance was evaluated using a Bonferroni-adjusted threshold of *p* < 0.0167 (0.05/3). *p*-value ^a^: Overall comparison among the three groups; *p*-value ^b^: non-severe PE vs. control; *p*-value ^c^: severe PE vs. control *p*-value ^d^: non-severe PE vs. severe PE.

**Table 2:** Comparison of laboratory parameters among the control, non-severe PE, and severe PE groups.

	Group 1 (Control) (n = 104) Median (IQR)	Group 2 (Non-Severe PE) (n = 70) Median (IQR)	Group 3 (Severe PE) (n = 31) Median (IQR)	*p*-Value ^a^	*p*-Value ^b^	*p*-Value ^c^	*p*-Value ^d^
ALT (U/L)	15.0 (5.0)	17.0 (14.0)	19.0 (8.0)	0.001	0.001	0.016	0.585
AST (U/L)	14.0 (6.0)	15.5 (8.5)	16.0 (6.0)	0.007	0.012	0.009	0.704
Platelet (10^9^/L)	244.0 (72.7)	226.0 (36.0)	226.0 (28.0)	0.023	0.212	0.008	0.324
Creatinine (mg/dL)	0.50 (0.10)	0.56 (0.12)	0.55 (0.18)	0.002	0.079	<0.001	0.696
Albumin (g/L)	38.0 (3.0)	36.0 (4.0)	36.0 (3.0)	<0.001	<0.001	<0.001	0.312
Fibrinogen (g/L)	—	4.9 (1.2)	5.5 (1.3)	—	—	—	0.003
FAR	—	0.14 (0.03)	0.16 (0.04)	—	—	—	0.012
PAR	6.33 (1.93)	6.24 (1.10)	6.57 (1.45)	0.570	0.538	0.638	0.243
CrAR	0.0132 (0.00)	0.0157 (0.00)	0.0151 (0.00)	<0.001	0.002	<0.001	0.680

Abbreviations: ALT, alanine aminotransferase; AST, aspartate aminotransferase; FAR, fibrinogen-to-albumin ratio; PAR, platelet-to-albumin ratio; CrAR, creatinine-to-albumin ratio; IQR, interquartile range; PE, preeclampsia. Statistical analysis: Overall comparisons among the three groups were performed using the Kruskal–Wallis test, and pairwise comparisons were performed using the Mann–Whitney U test. A *p*-value < 0.05 was considered statistically significant for overall comparisons. For the three pairwise comparisons of continuous variables, statistical significance was evaluated using a Bonferroni-adjusted threshold of *p* < 0.0167 (0.05/3). *p*-value ^a^: Overall comparison among the three groups; *p*-value ^b^: non-severe PE vs. control; *p*-value ^c^: severe PE vs. control *p*-value ^d^: non-severe PE vs. severe PE.

**Table 3:** ROC curve analysis of laboratory parameters and derived indices for discriminating severe PE from non-severe PE.

Parameter	Area	Standard Error	*p*-Value	AUC 95% CI Lower	AUC 95% CI Upper	Cut-Off Value	PPV/NPV (%)	LR+/ LR−	Sensitivity (%)	Specificity (%)
Platelet	0.562	0.062	0.324	0.441	0.682	—	—	—	—	—
Albumin	0.437	0.059	0.317	0.321	0.554	—	—	—	—	—
Creatinine	0.476	0.065	0.696	0.348	0.603	—	—	—	—	—
Fibrinogen	0.685	0.059	0.003	0.571	0.800	5.05	42.0/80.4	1.64/0.55	67.7	58.6
FAR	0.657	0.061	0.012	0.539	0.776	0.143	45.7/81.8	1.90/0.50	67.7	64.3
CrAR	0.474	0.064	0.680	0.348	0.600	—	—	—	—	—

Abbreviations: AUC, area under the curve; CI, confidence interval; FAR, fibrinogen-to-albumin ratio; CrAR, creatinine-to-albumin ratio; PE, preeclampsia. Cut-off values were reported only for parameters with statistically significant ROC performance. A *p*-value < 0.05 was considered statistically significant. PPV, positive predictive value; NPV, negative predictive value; LR+, positive likelihood ratio; LR−, negative likelihood ratio.

**Table 4:** Univariable logistic regression analysis of factors associated with severe PE.

Parameter	B	Standard Error	OR	95% CI for OR	*p*-Value
Fibrinogen	0.797	0.273	2.220	1.301–3.787	0.003
FAR × 100	0.185	0.072	1.203	1.044–1.386	0.011
CrAR × 1000	−0.024	0.054	0.976	0.878–1.085	0.654

Abbreviations: FAR, fibrinogen-to-albumin ratio; CrAR, creatinine-to-albumin ratio; OR, odds ratio; CI, confidence interval. FAR × 100 represents a 0.01-unit increase in the original FAR value, whereas CrAR × 1000 represents a 0.001-unit increase in the original CrAR value.

**Table 5:** Multivariable logistic regression analysis of factors independently associated with severe PE.

	Model 1 (Fibrinogen), aOR (95% CI), *p*-Value	Model 2 (FAR), aOR (95% CI), *p*-Value	Model 3 (CrAR), aOR (95% CI), *p*-Value
Age (years)	0.976 (0.875–1.090), 0.670	0.980 (0.881–1.091), 0.716	1.005 (0.907–1.115), 0.918
BMI (kg/m^2^)	0.937 (0.831–1.055), 0.282	0.957 (0.852–1.074), 0.455	0.953 (0.847–1.073), 0.431
Gestational age at sampling	1.184 (1.008–1.390), 0.039	1.186 (1.013–1.388), 0.034	1.247 (1.064–1.462), 0.006
Fibrinogen	2.242 (1.259–3.992), 0.006	—	—
FAR × 100	—	1.193 (1.025–1.389), 0.023	—
CrAR × 1000	—	—	0.930 (0.831–1.041), 0.208

Abbreviations: aOR, adjusted odds ratio; CI, confidence interval; BMI, body mass index; FAR, fibrinogen-to-albumin ratio; CrAR, creatinine-to-albumin ratio. Model 1 was adjusted for age, BMI, and gestational age at sampling and included fibrinogen; Model 2 was adjusted for age, BMI, and gestational age at sampling and included FAR × 100; Model 3 was adjusted for age, BMI, and gestational age at sampling and included CrAR × 1000. FAR × 100 represents a 0.01-unit increase in the original FAR value, whereas CrAR × 1000 represents a 0.001-unit increase in the original CrAR value.

## Data Availability

The data presented in this study are available on request from the corresponding author. The data are not publicly available due to privacy and ethical restrictions.

## Abbreviations

ACOG	American College of Obstetricians and Gynecologists
ACR	Albumin-to-creatinine ratio
ALT	Alanine aminotransferase
aOR	Adjusted odds ratio
AST	Aspartate aminotransferase
AUC	Area under the curve
BMI	Body mass index
CI	Confidence interval
CrAR	Creatinine-to-albumin ratio
C/S	Cesarean section
DM	Diabetes mellitus
FAR	Fibrinogen-to-albumin ratio
IQR	Interquartile range
NICU	Neonatal intensive care unit
OR	Odds ratio
PAR	Platelet-to-albumin ratio
PE	Preeclampsia
PPROM	Preterm prelabor rupture of membranes
ROC	Receiver operating characteristic
